# Acute Knockdown of Kv4.1 Regulates Repetitive Firing Rates and Clock Gene Expression in the Suprachiasmatic Nucleus and Daily Rhythms in Locomotor Behavior

**DOI:** 10.1523/ENEURO.0377-16.2017

**Published:** 2017-05-23

**Authors:** Tracey O. Hermanstyne, Daniel Granados-Fuentes, Rebecca L. Mellor, Erik D. Herzog, Jeanne M. Nerbonne

**Affiliations:** 1 Departments of Developmental Biology and Internal Medicine Washington University School of Medicine, Washington University, St. Louis, MO 63130; 2 Department of Biology, Washington University, St. Louis, MO 63130

**Keywords:** A-type current, action potential waveforms, Kv channels, Period2, repetitive firing properties, SCN

## Abstract

Rapidly activating and inactivating A-type K^+^ currents (I_A_) encoded by Kv4.2 and Kv4.3 pore-forming (α) subunits of the Kv4 subfamily are key regulators of neuronal excitability. Previous studies have suggested a role for Kv4.1 α-subunits in regulating the firing properties of mouse suprachiasmatic nucleus (SCN) neurons. To test this, we utilized an RNA-interference strategy to knockdown Kv4.1, acutely and selectively, in the SCN. Current-clamp recordings revealed that the *in vivo* knockdown of Kv4.1 significantly (*p* < 0.0001) increased mean ± SEM repetitive firing rates in SCN neurons during the day (6.4 ± 0.5 Hz) and at night (4.3 ± 0.6 Hz), compared with nontargeted shRNA-expressing SCN neurons (day: 3.1 ± 0.5 Hz; night: 1.6 ± 0.3 Hz). I_A_ was also significantly (*p* < 0.05) reduced in Kv4.1-targeted shRNA-expressing SCN neurons (day: 80.3 ± 11.8 pA/pF; night: 55.3 ± 7.7 pA/pF), compared with nontargeted shRNA-expressing (day: 121.7 ± 10.2 pA/pF; night: 120.6 ± 16.5 pA/pF) SCN neurons. The magnitude of the effect of Kv4.1-targeted shRNA expression on firing rates and I_A_ was larger at night. In addition, Kv4.1-targeted shRNA expression significantly (*p* < 0.001) increased mean ± SEM nighttime input resistance (R_in_; 2256 ± 166 MΩ), compared to nontargeted shRNA-expressing SCN neurons (1143 ± 93 MΩ). Additional experiments revealed that acute knockdown of Kv4.1 significantly (*p* < 0.01) shortened, by ∼0.5 h, the circadian period of spontaneous electrical activity, clock gene expression and locomotor activity demonstrating a physiological role for Kv4.1-encoded I_A_ channels in regulating circadian rhythms in neuronal excitability and behavior.

## Significance Statement

Neurons in the suprachiasmatic nucleus (SCN) use a transcription-translation feedback loop to generate daily changes in input resistances (R_in_) and firing rates that drive rhythms in physiology and behavior, although the molecular determinants underlying the daily changes in membrane properties have not been identified. We show here that Kv4.1 contributes to the generation of I_A_ and the regulation of the circadian period of electrical activity and clock gene expression in SCN neurons as well as locomotor behavior. These observations provide the first demonstration of a physiologic role for Kv4.1-encoded I_A_ channels in the mammalian brain. In addition, we show that the effects of Kv4.1 knockdown on I_A_, R_in_, and repetitive firing rates are greater at night than during the day.

## Introduction

Voltage-gated K^+^ (K_v_) channels are key regulators of neuronal excitability, functioning to control resting membrane potentials (V_r_), action potential waveforms, repetitive firing rates, as well as to modulate neurotransmitter release and synaptic plasticity (Rudy, 1988; Pongs, 1999). Multiple types of neuronal K_v_ channels with distinct time- and voltage-dependent properties and functional roles have been identified (Pongs, 1999; Gutman et al., 2005). Rapidly activating and inactivating A-type K_v_ currents (I_A_), for example, are widely expressed in the mammalian central nervous system (Rogawski, 1985; Rudy, 1988; Hoffman et al., 1997; Jerng et al., 2004), and several studies have linked Kv4.2 and Kv4.3, pore-forming (α) subunits of the Kv4 subfamily (Baldwin et al., 1991; Serodio et al., 1996) to the generation of I_A_. Electrophysiological experiments on hippocampal pyramidal CA1 neurons, for example, revealed a prominent role for Kv4.2 in shaping dendritic action potential waveforms (Kim et al., 2005). In cortical pyramidal neurons, however, both Kv4.2 and Kv4.3 contribute to the generation of I_A_ and the regulation of excitability (Carrasquillo et al., 2012). Although *in situ* hybridization studies have revealed that the third member of the Kv4 subfamily, Kv4.1, is also expressed in the rodent brain, the level of expression of Kv4.1 is low, in most brain regions, particularly when compared with Kv4.2 or Kv4.3 (Serodio and Rudy, 1998).

In the suprachiasmatic nucleus (SCN), the hypothalamic structure that controls circadian rhythms in mammalian physiology and behavior (Kalsbeek and Buijs, 2002; Colwell, 2011), I_A_ has been shown to be expressed and to modulate neuronal firing properties (Bouskila and Dudek, 1995; Alvado and Allen, 2008; Itri et al., 2010; Granados-Fuentes et al., 2012; Granados-Fuentes et al., 2015). Voltage-clamp recordings from SCN neurons in slices prepared from mice harboring targeted disruptions in *Kcnd2* (Kv4.2) or *Kcnd3* (Kv4.3) revealed a role for Kv4.2, but not Kv4.3, in the generation of I_A_ (Granados-Fuentes et al., 2012). It has, however, also been reported that Kv4.1 is expressed in the SCN, suggesting a functional role for Kv4.1 in the generation of I_A_ and in the regulation of SCN timing (Itri et al., 2010). To test this hypothesis directly, we developed an RNA-interference based strategy to examine the effects of acute, *in vivo* knockdown of Kv4.1 in the SCN. Whole-cell patch-clamp recordings from SCN cells expressing the Kv4.1-targeted shRNA revealed that knockdown of Kv4.1 expression increased repetitive firing rates and reduced macroscopic I_A_. Additional experiments revealed that acute knockdown of Kv4.1 shortened circadian periodicity in electrical activity, clock gene expression and locomotor activity.

## Methods and Materials

All reagents were obtained from Sigma-Aldrich, unless otherwise noted.

### Animals

All procedures involving animals were approved by the Animal Care and Use Committee and were conducted in accordance with the United States National Institutes of Health Guidelines for the Care and Use of Laboratory Animals. Mice were maintained on a C57Bl/6JN background (WT) in the Danforth and Medical School animal facilities. The *Per2^Luc^* mouse line, generated by replacing the endogenous mouse *Per2* locus with a PERIOD2::LUCIFERASE (PER2::LUC) reporter construct (Yoo et al., 2004), was obtained from Dr. J. Takahashi (University of Texas Southwestern, Dallas, TX).

### shRNA screening and validation of Kv4.1-targeted knockdown specificity

Several shRNA sequences targeting Kv4.1 were obtained from the RNAi Consortium and screened to determine the efficacy in reducing Kv4.1 expression. For screening, tsA201 cells were cotransfected using PepMute (Signagen) with a cDNA construct encoding Kv4.1-eYFP (obtained from A. Butler, Washington University) and one of the Kv4.1-targeted shRNAs or the nontargeted control shRNA (5’-CAACAAGATGAAGAGCACCAA-3’), which targets a variant of green fluorescent protein (turboGFP). Approximately 48 h later, cell lysates were prepared, fractionated by SDS-PAGE, transferred to polyvinylidene fluoride (PVDF) membranes and probed for GFP (Millipore, polyclonal anti-GFP, 1:1000) and Kv4.1 (Abcam polyclonal anti-Kv4.1, 1:500). Blots were also probed with an α-tubulin antibody (Abcam; monoclonal anti-α-tubulin, 1:10,000) to verify equal protein loading. The efficiency of the knockdown of Kv4.1-eGFP by each Kv4.1-targeted shRNA was quantified by densitometry. The shRNA sequence (5’-GCGGAGTGTGATGAGCCTTAT-3’) producing the largest reduction of Kv4.1-eGFP expression in tsA-201 cells was also evaluated for specificity. In these experiments, tsA201 cells were cotransfected with cDNA constructs encoding Kv4.1-eYFP, Kv4.2-eYFP, or Kv4.3 and either the Kv4.1-targeted shRNA or the nontargeted shRNA. Approximately 48 h after the transfections, lysates were fractionated by SDS-PAGE, transferred to PVDF membranes and probed for Kv4.1 (Abcam, polyclonal anti-Kv4.1, 1:500), Kv4.2 (NeuroMab, monoclonal anti-Kv4.2, 1:500) or Kv4.3 (NeuroMab, monoclonal anti-Kv4.3, 1:500).

The Kv4.1-targeted and the nontargeted shRNA 21-nucleotide sense sequences were synthesized into the corresponding 97-nucleotide miRNA-adapted shRNA oligonucleotides, containing sense and antisense sequences linked by a hairpin loop. Forward and reverse strands were annealed and cloned, in a microRNA (human miR30) context, in the 3’-untranslated region of eGFP in the pPRIME vector (Norris et al., 2010). The entire eGFP-shRNA cassette was then inserted into a viral shuttle vector with a synapsin (SYN) promoter and adeno-associated virus serotype 8 (AAV8) was generated by the Hope Center Virus Core Facility.

### Stereotaxic virus injections in the SCN

Under sterile conditions, adult (four- to nine-week) male WT C57BL/6 mice were anesthetized with isofluorane and secured in a stereotaxic head frame (Kopf Instruments). Eye ointment was applied to keep the eyes moist during the surgery. Heads were shaved, and Betadine was applied to cleanse and sterilize the shaved region. An incision was made along the midline and the skin was pulled back to expose the skull. For acute knockdown experiments, 1.2 μl of the nontargeted shRNA- or the Kv4.1-targeted shRNA-expressing AAV8 was injected into each side of the bilateral SCN (coordinates: 0.3 mm rostral to bregma, 0.1 mm left and right to midline, and 5.6 mm ventral to pial surface). The injection syringe (Hamilton) delivered the virus at a constant rate of 0.1 μl/min using a syringe pump (KD Scientific). The syringe was left in place for ∼5 min after the injection was complete to minimize the upward reflux of the solution during the removal of the needle. Silk sutures were used to close the incision. Animals were given an intraperitoneal injection of Rimadyl (0.1 ml of 0.05 mg/ml, Pfizer) and allowed to recover from the anesthesia on a heating pad maintained at 37°C.

### Preparation of acute SCN slices

Acute SCN slices (300 μm) were prepared from adult (6- to 12-week) male mice maintained in either a standard (lights on at 7 A.M. and lights off at 7 P.M.) or a reversed 12/12 h light/dark (LD) cycle (Granados-Fuentes et al., 2012). Zeitgeber times (ZTs) are indicated: ZT0 corresponds to the time of lights on and ZT12 to the time of lights off in the animal facility. Daytime slices were routinely prepared at ZT5 from mice maintained in the standard LD cycle and nighttime slices were prepared at ZT15 from mice maintained in a reversed (i.e., lights on at 7 P.M. and lights off at 7 A.M.) LD cycle. For the preparation of daytime slices, brains were rapidly removed (in the light) from animals anesthetized with 1.25% Avertin (Acros Organics, 2,2,2-tribromoethanol and tert-amyl alcohol in 0.9% NaCl; 0.025 mL/g body weight) and placed in ice-cold cutting solution containing: 240 mM sucrose, 2.5 mM KCl, 1.25 mM NaH_2_PO_4_, 25 mM NaHCO_3_, 0.5 mM CaCl_2_, and 7 mM MgCl_2_, saturated with 95% O_2_/5% CO_2_. For the preparation of nighttime slices, animals in the reversed LD cycle were removed from their cages at ZT15 under infrared illumination (to avoid exposure to visible light during the preparation of acute SCN slices), anesthetized with isoflorane and enucleated using previously described procedure (Aton et al., 2004; Hattar et al., 2006; Hermanstyne et al., 2016). Following an intraperitoneal injection of Rimadyl (0.1 ml of 0.05 mg/ml, Pfizer), each animal was allowed to recover from the anesthesia (for ∼1 h) before transport to the laboratory for the preparation of slices. At ZT16, animals were anesthetized with 1.25% Avertin; brains were rapidly removed and placed in ice-cold cutting solution. For all experiments, coronal slices (300 µm) were cut on a Leica VT1000 S vibrating blade microtome (Leica Microsystems) and incubated in a holding chamber with oxygenated artificial cerebrospinal fluid (ACSF) containing: 125 mM NaCl, 2.5 mM KCl, 1.25 mM NaH_2_PO_4_, 25 mM NaHCO_3_, 2 mM CaCl_2_, 1 mM MgCl_2_, and 25 mM dextrose (∼310 mOsmol l^−1^), saturated with 95% O_2_/5% CO_2_, at room temperature (23–25°C) for at least 1 h before transfer to the recording chamber.

### Electrophysiological recordings

Whole-cell voltage- and current-clamp recordings were obtained from SCN neurons in slices prepared during the day (ZT7–ZT12) or at night (ZT19–ZT24) at room temperature (23–25°C) from WT animals and from animals injected with the nontargeted shRNA- or the Kv4.1-targeted shRNA-expressing AAV8. SCN neurons were visually identified in slices using differential interference contrast optics with infrared illumination. Slices were perfused continuously with ACSF saturated with 95% O_2_/5% CO_2_. For voltage-clamp recordings, the ACSF also contained tetraethylammonium (3 mM), CdCl_2_ (0.1 mM), and tetrodotoxin (150 nM). Recording pipettes (3–5 MΩ) contained: 144 mM K-gluconate, 10 mM HEPES, 3 mM MgCl_2_, 4 mM MgATP, 0.2 mM EGTA, and 0.5 mM NaGTP (pH 7.3; 300 mOsmol l^−1^). Voltage-clamp paradigms were generated and data were collected using a Multiclamp 700B patch clamp amplifier (Molecular Devices) interfaced to a Dell personal computer with a Digidata 1332 and the pCLAMP 10 software package (Molecular Devices). Tip potentials were zeroed before membrane-pipette seals were formed. Following formation of a GΩ seal and establishing the whole-cell configuration, membrane capacitances and series resistances were compensated electronically. Series resistances before compensation were in the range of 15–20 MΩ, and were routinely corrected by 70–80%. If the series resistance changed ≥20% during the recording, the experiment was stopped and acquired data from that cell were not included in the analyses. Voltage signals were acquired at 20 kHz, filtered at 10 kHz, and stored for offline analysis. Whole-cell membrane capacitances were determined from analyses of capacitative currents elicited by brief (25 ms) voltage steps (±20 mV) from the holding potential (−70 mV).

Rapidly activating and inactivating Kv currents, I_A_, were isolated using a two-step voltage-clamp protocol (Norris and Nerbonne, 2010; Granados-Fuentes et al., 2012; Granados-Fuentes et al., 2015). Briefly, in each cell, whole-cell Kv currents, evoked in response to 2 s depolarizing voltage steps to potentials between −40 and +30 mV (in 10-mV increments) from a holding potential of −70 mV, were first recorded. Currents were then recorded again with a prepulse paradigm that included a brief (60 ms) step to −10 mV before the 2 s depolarizing voltage steps to potentials between −40 and +30 mV (in 10-mV increments). Off-line subtraction of the Kv currents recorded with the prepulse paradigm from the currents evoked without the prepulse provided I_A_.

In separate experiments, the voltage dependences of activation and steady-state inactivation of I_A_ in nontargeted shRNA- and Kv4.1-targeted shRNA-expressing SCN neurons were examined. To generate the activation plots, I_A_ was isolated using a two-step protocol, as described above. In these experiments, however, Kv currents were evoked in response to test potentials from −65 mV to +35 mV (in 5-mV increments) from a holding potential of −70 mV. In each cell, I_A_ conductances (G_IA_) at each test potential were calculated and normalized to the maximal conductance (G_IAmax_). Mean ± SEM normalized I_A_ conductances (G_IA_/G_IAmax_) were plotted as a function of the test potential and fitted using the Boltzmann equation, G_IA_/G_IA,max_ = 1 + *e* ^[(Va – Vm)/k]^, where V_a_ is the membrane potential of half-maximal activation and *k* is the slope factor.

To determine the voltage dependence of steady-state inactivation of I_A_, a three-step voltage-clamp paradigm was used. In each cell, Kv currents evoked at +10 mV from different conditioning voltages, ranging from −120 to −20 mV (in 5-mV increments). In each cell, I_A_ evoked from each conditioning voltage was measured and normalized to the maximum current evoked from the most hyperpolarized (−100 mV) membrane potential (in the same cell). Mean ± SEM normalized current amplitudes (I_A_/I_Amax_) were plotted as a function of the conditioning voltage and fitted using the Boltzmann equation, I_A_/I_A,max_ = 1/(1 + *e* ^[(Vh – Vm)/k]^), where V_h_ in the membrane potential at half-maximal inactivation and *k* is the slope factor.

Whole-cell current-clamp recordings were obtained using pipettes (4–7 MΩ) containing: 144 mM K-gluconate, 10 mM HEPES, 3 mM MgCl_2_, 4 mM MgATP, 0.2 mM EGTA, and 0.5 mM NaGTP (pH 7.3; 300 mOsm). Slices were perfused continuously with ACSF containing 20 μM Gabazine (Tocris Bioscience) and saturated with 95% O_2_/5% CO_2_. A loose patch, cell-attached recording was first obtained and spontaneous activity was recorded for ∼1 min (Hermanstyne et al., 2016). Following the formation of a GΩ seal, the whole-cell configuration was established and whole-cell membrane capacitances and series resistances were compensated. Whole-cell spontaneous firing activity was then recorded for ∼1 min. Access resistances were 15–20 MΩ, and data acquisition was terminated if the access resistance increased (20%) during the experiment. Voltage signals were acquired at 100 kHz, filtered at 10 kHz and stored for offline analysis. Input resistances (R_in_) were determined by measuring the steady-state voltage changes produced by ± 5 pA current injections from a hyperpolarized membrane potential. The voltage threshold for action potential generation (APT) in each cell was determined as the point during the upstroke (depolarizing phase) of the action potential at which the second derivative of the voltage was zero. Afterhyperpolarization amplitudes (AHPs) were measured in each cell as the difference between the APT and the most negative membrane potential. Action potential durations were measured at 50% repolarization (APD_50_). V_r_ were determined from phase plots of the first derivative of the membrane potential (dV/dT) plotted versus the membrane potential (mV). Statistical analyses were performed using one-way ANOVA with Newman-Kuels *post hoc* pairwise comparisons or two-sample Kolmogorov-Smirnov test. All data were analyzed using GraphPad Prism software with the exception of the cumulative distribution plots, which were analyzed using OriginLab with the two-sample Kolmogorov-Smirnov test. Statistical significance was set at *P* < 0.05; *P* values are reported in the text, Table 1 and figure legends. The structure of the dataset and the statistical power for each analysis are provided in Table 2. The letters (a–m) in Table 2 represent the statistical analyses performed and correspond to the superscripts presented in the Results section. 

**Table 1.**
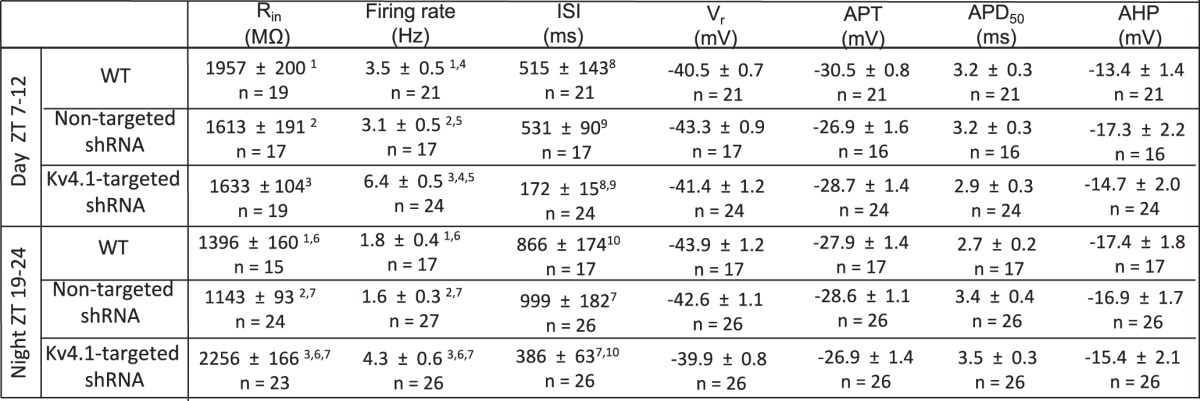
Resting and active membrane properties of WT, nontargeted-, and Kv4.1-targeted shRNA-expressing SCN neurons during the day and at night

Values are means ± SEM; *n*, number of cells; R_in_, input resistance; ISI, interspike interval; V_r_, resting membrane potential; APT, action potential threshold; APD_50_, action potential duration at 50% repolarization; AHP, afterhyperpolarization amplitude. ^1^Values in WT neurons during the day and at night (^1^*p* < 0.05) are significantly different. ^2^Values in nontargeted shRNA-expressing neurons during the day and at night (^2^*p* < 0.05) are significantly different. ^3^Values in Kv4.1-targeted shRNA-expressing neurons during the day and at night (^3^*p* < 0.01) are significantly different. ^4–9^Values in WT and nontargeted shRNA-expressing neurons during the day (^4^*p* < 0.0006; ^5^*p* < 0.0001; ^8,9^*p* < 0.05) are significantly different from Kv4.1-targeted shRNA-expressing neurons and at night (^6^*p* < 0.0006; ^7^*p* < 0.0001; ^10^*p* < 0.01).

**Table 2.** Statistical table

	Data structure	Type of test	Power
a	Normal distribution	One-way ANOVA	*F* statistic: 14.2
b	Normal distribution	One-way ANOVA	*F* statistic: 7.54
c	Normal distribution	One-way ANOVA	*F* statistic: 52.15
d	Normal distribution	One-way ANOVA	*F* statistic: 1.99
e	Normal distribution	One-way ANOVA	*F* statistic: 0.53
f	Normal distribution	One-way ANOVA	*F* statistic: 1.11
g	Normal distribution	One-way ANOVA	*F* statistic: 0.37
h	Normal distribution	One-way ANOVA	*F* statistic: 17.9
i	Continuous distribution	Kolmogorov-Smirnov	*D* statistic: 0.4
j	Non-normal distribution	Mann-Whitney-Wilcoxon test	*U* statistic: 819
k	Normal distribution	One-way ANOVA	*F* statistic: 4.03
l	Normal distribution	Student’s *t* test	*T* statistic: 5.68
m	Normal distribution	Student’s *t* test	*T* statistic: 3.04
n	Continuous distribution	Kolmogorov-Smirnov	D statistic: 0.6

### Extracellular multielectrode array recordings

Dispersed SCN neuron cultures were prepared as previously described (Herzog et al., 1998). Briefly, SCN were removed from postnatal day 4 (P4) PER2^LUC^ mice, and (300 µm) coronal slices were cut on a Vibratome (OTS-5000; Electron Microscopy Sciences). Four to six SCN punches (1 mm diameter) were taken from each slice and enzymatically dissociated using papain. Isolated neurons were plated at a density of 10,000 neurons/mm^2^ on poly-D-lysine and laminin-coated multielectrode arrays (sixty 30-µm- diameter electrodes, Multichannel Systems). Cultures were maintained in 1 ml Dulbeccos Modified Eagles Medium (DMEM) supplemented with 10% fetal calf serum (Life Technologies) at 37°C in a 95% O_2_/5% CO_2_ incubator. For knockdown experiments, cultures were incubated with the nontargeted shRNA- or the Kv4.1-targeted shRNA-expressing AAV8 for 3 d. Extracellular action potential recordings were obtained from dispersed SCN neurons for at least 5 d, as previously described (Aton et al., 2005). Single-cell action potentials were digitized in real-time (MC-Rack Software, Multichannel Systems) and discriminated offline using principal component analysis (Offline Sorter). NeuroExplorer software was used to bin the firing rates in 10 min intervals. Firing rate rhythms were fitted with a damped sine function (Chronostar 2.0; Maier et al., 2009) and data with correlation coefficients ≥ 0.8 and a period of 18–32 h were defined as circadian. Repetitive firing rates at the peak and trough of activity in Kv4.1 shRNA- and nontargeted shRNA-expressing SCN neurons were averaged over 3 d and compared using one-way ANOVA with Newman-Kuels *post hoc* pairwise comparisons.

### Real-time gene expression (PER2::LUC) recordings

Bioluminescence was recorded from SCN slices prepared from P4 PER2^LUC^ mice housed in a standard 12:12 h LD cycle. For the preparation of slices, brains were quickly removed and chilled in Hanks’ balanced salt solution (HBSS), supplemented with 0.01 M HEPES, 100 U/ml penicillin, 0.1 mg/ml streptomycin, and 4 mM NaHCO_3_. Vibratome slices (300 μm) were cut and placed on 0.4 mm membrane inserts (Millipore) in 35-mm Petri dishes (BD Biosciences) with 1-ml HEPES-buffered DMEM supplemented with 10% newborn calf serum (Invitrogen) and 0.1 mM beetle luciferin (Biosynth). Immediately after plating, slices were transduced with either the Kv4.1-targeted shRNA- or the nontargeted shRNA-expressing AAV8. Virus-containing media was removed after 3 d. After 2 weeks in culture, Petri dishes were sealed with vacuum grease and placed under photomultiplier tubes (HC135-11MOD; Hamamatsu) at 36°C in the dark. Bioluminescence was recorded in 10-min bins for at least 5 d. The period of PER2^LUC^ expression was determined using Chronostar and compared using a one-way ANOVA followed by a Tukey *post hoc* test.

### Analyses of locomotor activity

Bilateral injections (1.2 μl total volume) of the nontargeted shRNA- or the Kv4.1 shRNA-expressing AAV8 were made in adult (8–12 week-old) WT male mice (during the day). Approximately 10 d after surgery, animals were placed individually in cages equipped with running wheels in light-tight chambers illuminated with fluorescent bulbs (2.4 ± 0.5 × 10^18^ photons/s*m^2^; General Electric). Wheel-running activity was recorded in 6-min bins (Clocklab Actimetrics) for 5–10 d in a 12:12 h LD cycle followed, by 15–20 d in constant darkness (DD). The period of behavioral rhythmicity of each mouse was determined using χ^2^ periodogram analysis (Sokolove and Bushell, 1978) from continuous recordings of 10 d in DD (Clocklab). Rhythmicity was considered statistically significant if the χ^2^ periodogram value exceeded the 99.9% confidence interval (Qp value).

## Results

### Efficient and specific shRNA-mediated knockdown of Kv4.1

**Figure 1.**
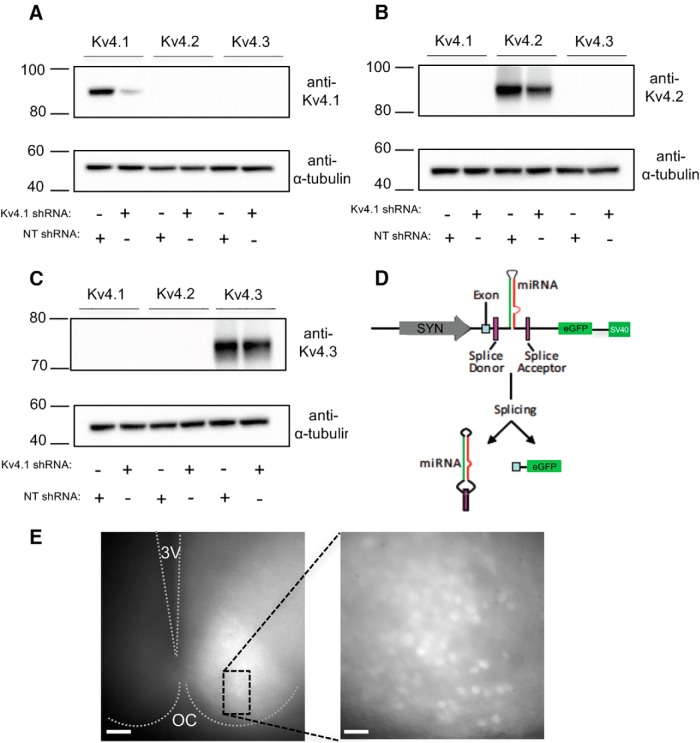
Validation of Kv4.1-targeted shRNAs. ***A–C***, The Kv4.1-eYFP, Kv4.2-eYFP, or Kv4.3 construct was coexpressed with either the Nontargeted (NT) shRNA or the Kv4.1-targeted shRNA in tsA-201 cells. Approximately 48 h later, lysates were prepared, fractionated by SDS-PAGE, transferred to PVDF membranes, and probed with an anti-Kv4.1, anti-Kv4.2, or anti-Kv4.3 antibody. Blots were also reprobed with an anti-α tubulin (α-tubulin) antibody to verify equal loading of proteins. The Kv4.1-targeted shRNA markedly reduced the expression of Kv4.1, compared with the nontargeted shRNA (***A***), whereas neither Kv4.2 (***B***) nor Kv4.3 (***C***) expression was measurably affected. ***D***, The Kv4.1-targeted and the nontargeted shRNAs were cloned, in a microRNA (miR30) context, between splice donor and acceptor sequences in an artificial intron downstream of the SYN promoter and upstream of the reporter (eGFP) cDNA (Norris et al., 2010). ***E***, An acute SCN slice, prepared from an adult mouse 27 d following a unilateral injection of the Kv4.1-targeted shRNA-expressing AAV8 into the right hemisphere, is shown. In the left panel, a low-magnification fluorescence image reveals eGFP expression in the right hemisphere only. The third ventricle (3V) and the optic chiasm (OC) are marked. In the higher magnification image on the right, virally-transduced eGFP-expressing neurons are evident throughout the SCN. Scale bars: 200 and 50 μm in the left and right panels, respectively.

An RNA interference-based strategy was developed to allow the acute *in vivo* knockdown of Kv4.1 in adult mouse SCN neurons. Several shRNA sequences targeted against Kv4.1 were screened for effectiveness in reducing Kv4.1 expression in tsA201 cells as described in Methods and Materials. The specificity of the shRNA that provided the greatest (∼80%) knockdown (see Methods and Materials) was also evaluated in tsA201 cells cotransfected with the Kv4.1-targeted shRNA or the nontargeted shRNA together with a cDNA construct encoding Kv4.1, Kv4.2 or Kv4.3. As illustrated in Figure 1, the Kv4.1-targeted shRNA was effective in reducing the expression of Kv4.1 (Fig. 1*A*), whereas neither Kv4.2 (Fig. 1*B*) nor Kv4.3 (Fig. 1*C*) expression was measurably affected by coexpression with the Kv4.1-targeted shRNA.

The Kv4.1-targeted shRNA and the nontargeted shRNA were cloned (separately), in a microRNA (human miR30) context, into a plasmid containing a SYN promoter and eGFP (Norris et al., 2010). Using this strategy (Fig. 1*D*), the shRNA and GFP are processed from a single transcript. For experiments, adeno-associated viruses serotype 8 (AAV8) were generated as described in Methods and Materials. Stereotaxic injections of the Kv4.1-targeted shRNA- or the nontargeted shRNA-expressing AAV8 were made into the SCN of 6- to 12-week-old mice. Transduced SCN neurons in acute SCN slices were visually identified by the presence of eGFP (Fig. 1*E*).

### Knockdown of Kv4.1 attenuates IA in SCN neurons

**Figure 2.**
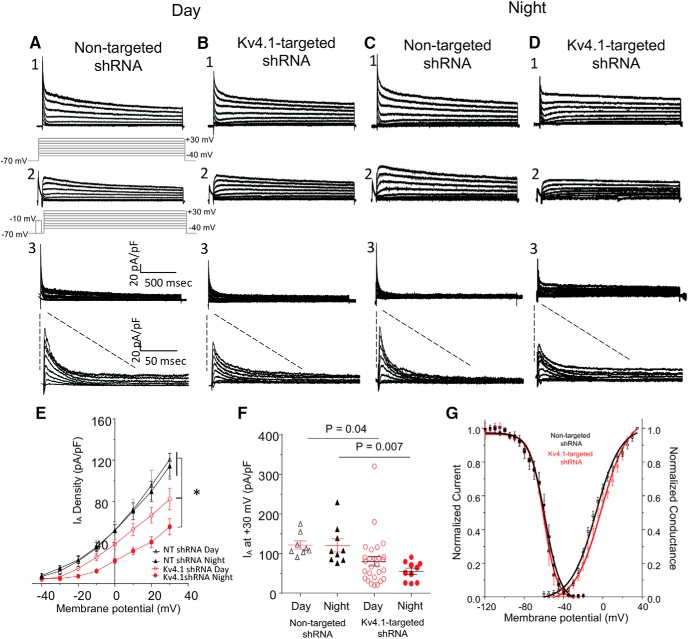
Acute knockdown of Kv4.1 decreases A-type K^+^ currents (I_A_) in SCN neurons during the day and at night. ***A–D***, Representative whole-cell Kv current recordings, obtained during the day (ZT7–ZT12) or at night (ZT19–ZT24) from eGFP-positive SCN neurons in acute slices prepared from adult animals 14–30 d following injections of the nontargeted shRNA-expressing (***A***, ***C***) or the Kv4.1-targeted shRNA-expressing (***B***, ***D***) AAV8, are shown. Whole-cell Kv currents, evoked during (2 s) voltage steps to potentials ranging from −40 to +30 mV (in 10-mV increments) from a holding potential of −70 mV, were recorded from nontargeted shRNA- and Kv4.1-targeted shRNA-expressing SCN neurons during the day (***A1***–***B1***) and at night (***C1***–***D1***). A second voltage-clamp paradigm, that included a brief (60 ms) prepulse to −10 mV to inactivate I_A_, was then presented, and whole-cell Kv currents in each cell were recorded again (***A2***–***D2***). The voltage-clamp paradigms are illustrated (light gray) below the current records. Digital offline subtraction of the recordings with the prepulse (***A2***–***D2***) from those without the prepulse (***A1***–***D1***) isolated I_A_ (***A3***–***D3***); the subtracted records are also shown on an expanded time scale. ***E***, I_A_ densities at each test potential in each cell were calculated and mean ± SEM values are plotted as a function o*f* test potential. ***F***, Mean ± SEM I_A_ densities at +30 mV were significantly lower in Kv4.1-targeted shRNA-expressing (*n* = 10–27), than in nontargeted shRNA-expressing (*n* = 8–10) SCN neurons during the day and at night; *p* values (one-way ANOVA) are indicated. ***G***, The activation curves for Kv4.1-targeted shRNA-expressing SCN neurons (open red squares, *n* = 8) and nontargeted shRNA-expressing SCN neurons (open black squares, *n* = 12) are not significantly (*p =* 0.052, Student’s *t* test) different (V_1/2_ = −1.8 ± 3.2 mV, *k* = 13.5 ± 1.1 and −9.3 mV ± 1.3 mV, *k* = 12.7 ± 0.8, respectively). The inactivation curves plotted as the mean ± SEM normalized current for Kv4.1-targeted shRNA-expressing (filled red squares, *n* = 8) and nontargeted shRNA-expressing (filled black squares, *n* = 12) SCN neurons are also shown. The inactivation curve for Kv4.1-targeted shRNA-expressing SCN neurons (V_1/2_ = −60.3 mV ± 1.7 mV and *k* = 7.0 ± 0.6) is not significantly (*p >* 0.05, Student’s *t* test) different from the nontargeted shRNA-expressing SCN neurons (V_1/2_ = −59.4 mV ± 2.1 mV and *k* = 7.9 ± 0.7).

Whole-cell voltage-clamp recordings were obtained from eGFP-expressing SCN neurons in acute slices prepared from animals injected with either the Kv4.1-targeted shRNA-or the nontargeted shRNA-expressing AAV8. Consistent with previous studies (Itri et al., 2010; Granados-Fuentes et al., 2012; Granados-Fuentes et al., 2015), I_A_ was readily identified in every cell examined (Fig. 2*A–D*). To quantify the amplitudes/densities of I_A_, a two-step voltage-clamp paradigm, which is illustrated below the current records in Fig. 2*A1*–*A2*, was used. Offline subtraction of the current records obtained with the prepulse from the controls provided I_A_ (see Methods and Materials). The amplitudes/densities of I_A_ in SCN neurons expressing the Kv4.1-targeted shRNA were ∼35% – 50% lower than those measured in SCN neurons transduced with the nontargeted shRNA-expressing AAV8 (Fig. 2*A3–D3*)^k^. At +30 mV, for example, mean I_A_ densities were significantly (*p* = 0.04) lower during the day and at night in Kv4.1-targeted shRNA-expressing SCN neurons (day: 80.3 ± 11.8 pA/pF; night: 55.3 ± 7.7 pA/pF) than in nontargeted shRNA-expressing SCN neurons (day: 121.7 ± 10.2 pA/pF; night: 120.6 ± 16.5 pA/pF; Fig. 2*F*)^k^. Interestingly, the knockdown of Kv4.1 resulted in a greater effect on I_A_ density at night than during day (see Discussion).

Additional experiments were completed (as described in Materials and Methods) to examine the voltage dependences of activation and steady-state inactivation of I_A_ in Kv4.1-targeted shRNA- and nontargeted shRNA-expressing SCN neurons. The mean ± SEM normalized data are presented in Figure 2*G*. The activation curves for I_A_ in Kv4.1-targeted shRNA- and nontargeted shRNA-expressing SCN neurons were similar. Note that, although the activation curve for I_A_ in Kv4.1-targeted shRNA-expressing SCN neurons appears to be shifted slightly compared with the nontargeted shRNA-expressing cells, the V_1/2_ activation and *k* values derived from the fits were not significantly (*p* = 0.052) different (Fig. 2*G*). The voltage dependences of steady-state inactivation of I_A_ in Kv4.1-targeted shRNA- and nontargeted shRNA-expressing SCN neurons (Fig. 2*G*) were also not significantly (*p >* 0.05) different (see Discussion).

### Acute knockdown of Kv4.1 increases spontaneous firing rates in SCN neurons

**Figure 3.**
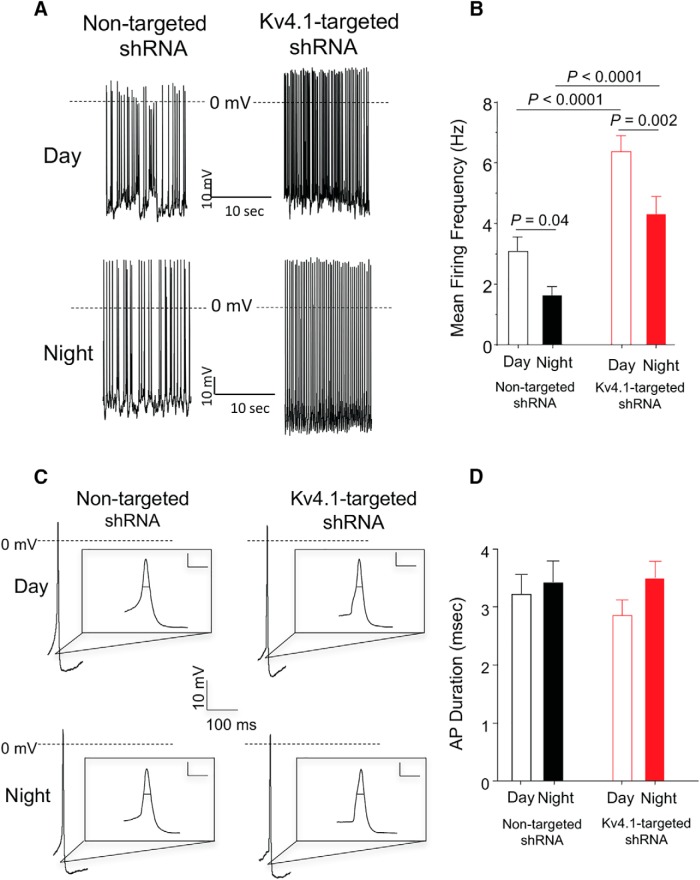
Acute knockdown of Kv4.1 increases spontaneous firing rates in SCN neurons during the day and at night. ***A***, Representative daytime (top) and nighttime (bottom) whole-cell current-clamp recordings obtained from nontargeted shRNA-expressing (left) and Kv4.1-targeted shRNA-expressing (right) SCN neurons are illustrated. ***B***, Mean ± SEM firing frequencies in both nontargeted shRNA-expressing (*n* = 17–27) and Kv4.1-targeted shRNA-expressing neurons (*n* = 24–26) are significantly higher during the day than at night. In addition, the mean ± SEM daytime and nighttime firing frequencies in Kv4.1-targeted shRNA-expressing SCN neurons (*n* = 24–26) are significantly higher than in nontargeted shRNA-expressing (*n* = 17–27) SCN neurons; *p* values (one-way ANOVA) are indicated. ***C***, Representative action potential waveforms recorded in nontargeted shRNA-expressing (left) and Kv4.1-targeted shRNA-expressing (right) SCN neurons during the day (top) and at night (bottom) are shown. The records are also illustrated on an expanded time scale in the insets (scale bar, 10 mV and 10 ms). ***D***, The mean ± SEM APD_50_ values determined in both nontargeted shRNA-expressing (*n* = 16–26) and Kv4.1-targeted shRNA-expressing neurons (*n* = 24–26) were not significantly (*p* > 0.05, one-way ANOVA) different during the day or at night (Table 1).

Whole-cell current-clamp recordings were obtained from eGFP-expressing SCN neurons in acute slices prepared from animals injected with either the Kv4.1-targeted shRNA- or the nontargeted shRNA-expressing AAV8. Similar to previous reports (Welsh et al., 1995; Pennartz et al., 2002; Hermanstyne et al., 2016), all of the SCN neurons examined were spontaneously active and repetitive firing rates measured in both Kv4.1-targeted shRNA- and nontargeted shRNA-expressing cells were significantly *(p* < 0.04) higher in recordings obtained during the day than at night (Fig. 3*A*,*B*)^a^. Importantly, additional recordings from nontransduced, i.e., non-eGFP-expressing, wildtype (WT) SCN neurons in the same slices revealed repetitive firing rates that were not significantly different from those determined in nontargeted shRNA-expressing SCN neurons (Table 1)^a^. The firing rates of SCN neurons transduced with the Kv4.1-targeted shRNA-expressing AAV8, however, were significantly *(p* < 0.0001) higher than SCN neurons transduced with the nontargeted shRNA-expressing AAV8 during the day and at night (Fig. 3*B* and Table 1)^a^.

Further analyses of the whole-cell current-clamp data revealed that, similar to previous findings in WT SCN neurons (Schaap et al., 1999; Pennartz et al., 2002), there was a marked *(p* < 0.05) day-night difference in the mean R_in_s of SCN neurons expressing the nontargeted shRNA (Table 1)^b^. Compared with nontargeted shRNA-expressing SCN neurons, the acute knockdown of Kv4.1 significantly *(p* < 0.0001) increased the mean R_in_ at night, but not during the day (Table 1)^b^, observations suggesting a physiologic role for Kv4.1-encoded channels in regulating the daily rhythms in the excitability of SCN neurons (see Discussion). In addition, during the day and at night, mean interspike intervals (ISIs) were significantly (*p* < 0.05) shorter in Kv4.1-targeted shRNA-expressing SCN neurons (Table 1)^c^. In contrast, the waveforms of individual action potentials recorded from Kv4.1-targeted shRNA- and nontargeted shRNA-expressing SCN neurons were similar (Fig. 3*C*). Mean APD_50_ in Kv4.1-shRNA- and nontargeted shRNA-expressing SCN neurons, for example, were not significantly (*p* > 0.05) different during the day and at night (Fig. 3*D* and Table 1). In addition, there were no significant differences between AHPs, APTs, or the V_r_ measured in Kv4.1-targeted shRNA- and nontargeted shRNA-expressing SCN neurons during the day or at night (Table 1)^d–g^.

### Acute knockdown of Kv4.1 alters circadian properties of spontaneous electrical activity in SCN neurons

**Figure 4.**
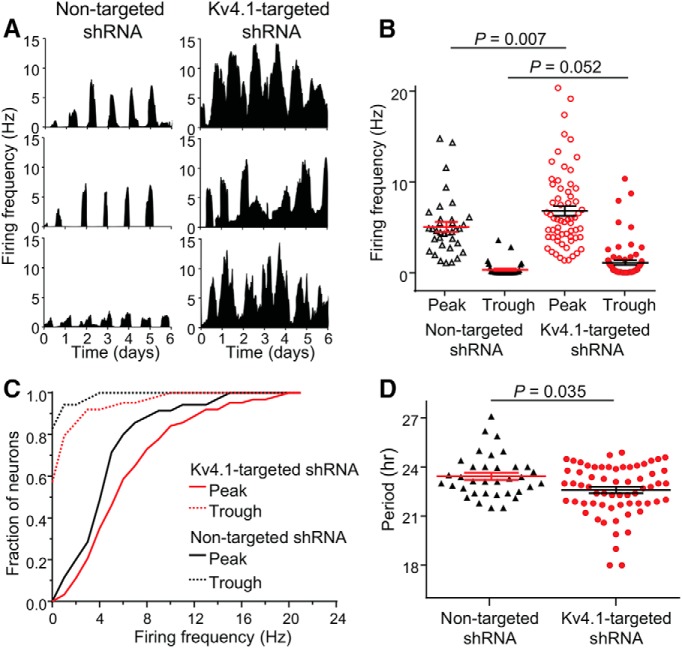
Acute knockdown of Kv4.1 alters the frequency and period of circadian firing in SCN neurons. Spontaneous firing was recorded continuously (over 6 d) from SCN neurons in dispersed cultures. ***A***, Representative recordings from three SCN neurons in cultures transduced with either the nontargeted shRNA-expressing (left) or the Kv4.1-targeted shRNA-expressing (right) AAV8 are shown. ***B***, The average peak and trough repetitive firing rates, measured in individual nontargeted shRNA-expressing (black open and filled triangles; *n* = 35) and Kv4.1-targeted shRNA-expressing (red open and filled circles; *n* = 63) SCN neurons are plotted; mean ± SEM values are indicated. As is evident, mean ± SEM peak firing rates were significantly higher in Kv4.1-targeted shRNA-expressing, compared with nontargeted shRNA-expressing, SCN neurons; *p* values (one-way ANOVA) are indicated. ***C***, Cumulative distribution plots of peak and trough firing rates reveal rightward shifts (toward higher frequencies) in Kv4.1-targeted shRNA-expressing, compared with nontargeted shRNA-expressing, SCN neurons (*p* < 0.05, two-sample Kolmogorov-Smirnov test). ***D***, The circadian periods of firing measured in individual nontargeted shRNA-expressing (*n* = 35) and Kv4.1-targeted shRNA-expressing (*n* = 63) SCN neurons are plotted; mean ± SEM values are indicated. The mean ± SEM period was significantly (*p* < 0.035; Mann-Whitney-Wilcoxon test) shorter in SCN neurons expressing the Kv4.1-targeted shRNA, compared with cells expressing the nontargeted shRNA.

In parallel with the *in vitro* slice recordings, we examined the functional consequences of the *in vitro* knockdown of Kv4.1 expression on circadian rhythms in repetitive firing in dispersed SCN neurons using multi-electrode arrays (see Methods and Materials). Continuous recordings of spontaneous firing were obtained over 6 d. As illustrated in Figure 4*A*, we observed rhythmic electrical activity in SCN neurons transduced with either the Kv4.1-targeted shRNA- or the nontargeted shRNA-expressing AAV8. In SCN neurons transduced with the Kv4.1 shRNA-expressing AAV8 (6.5 ± 0.6 Hz), however, the mean firing frequency was significantly (*p* = 0.007) higher during the peak (subjective day) of electrical activity, compared with nontargeted shRNA-expressing (5.2 ± 0.5 Hz) SCN neurons (Fig. 4*A*,*B*)^h^. During the trough (subjective night), the mean firing frequency was also higher in Kv4.1-targeted shRNA-expressing (1.1 ± 0.3 Hz) than in nontargeted shRNA-expressing (0.3 ± 0.1 Hz) SCN neurons (Fig. 4*A*,*B*)^h^.

The cumulative distribution plot reveals that, during the peak of electrical activity, ∼80% of the Kv4.1-targeted and nontargeted shRNA-expressing SCN neurons fired at <10 and 6 Hz, respectively (Fig. 4*C*)^i^. The vast majority (>80%) of nontargeted shRNA-expressing SCN neurons were electrically silent during the trough (Fig. 4*C*)^i^. In contrast, the majority of Kv4.1 shRNA-expressing SCN neurons fired repetitively at 1 Hz or higher during the trough (Fig. 4*C*)^i^. The mean circadian period was also significantly (*p =* 0.035) shorter in SCN neurons transduced with the Kv4.1-targeted shRNA-expressing (22.7 ± 0.2 h), compared with nontargeted shRNA-expressing (23.3 ± 0.2 h), AAV8 (Fig. 4*D*)^j^.

### Knockdown of Kv4.1 shortens the circadian period of clock gene expression

**Figure 5.**
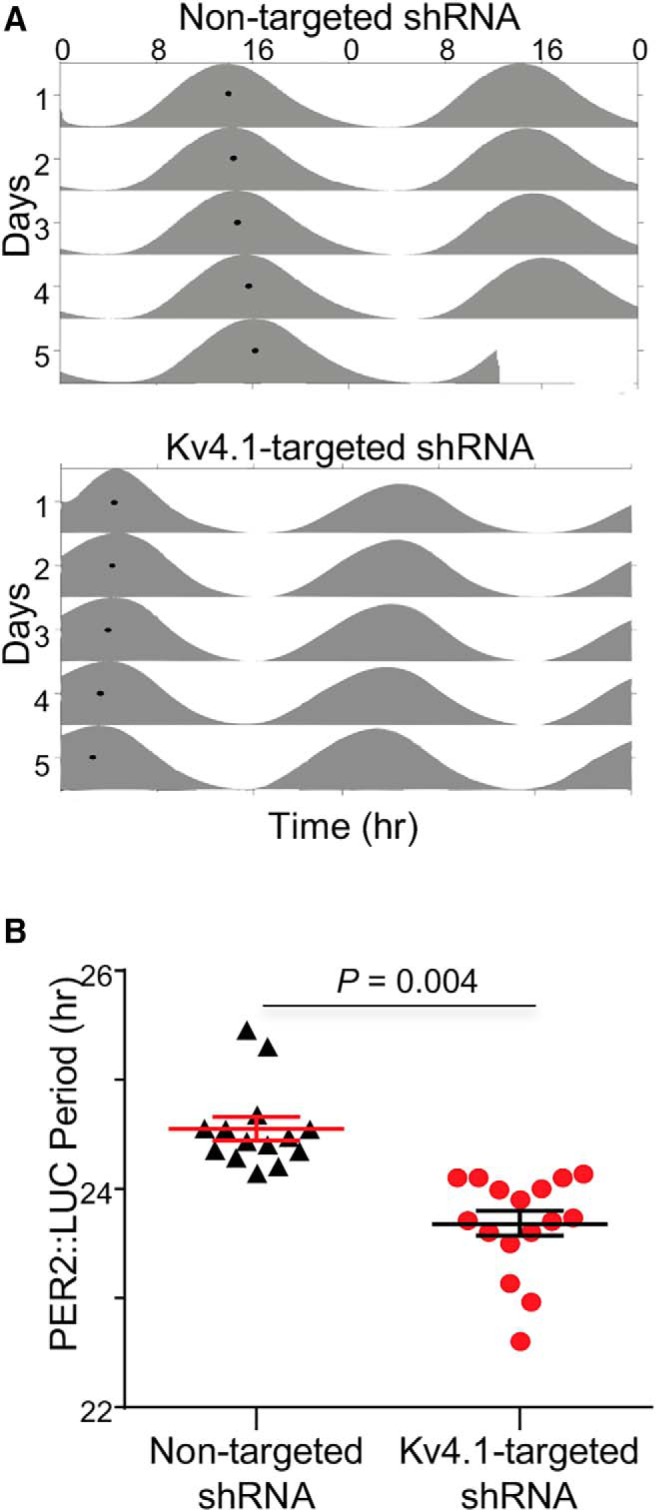
Acute knockdown of Kv4.1 shortens the circadian period of PER2^LUC^ expression in isolated SCN. ***A***, Representative PER2^LUC^ bioluminescence recordings, shown as double-plotted actograms, were obtained from isolated SCN infected with the nontargeted shRNA-expressing (top) or the Kv4.1-targeted shRNA-expressing (bottom) AAV8. The black dots on the left sides of each of the actograms represent the acrophase (the peak) of each rhythm. ***B***, The mean ± SEM circadian period was significantly (*p* < 0.004; Student’s *t* test) shorter in SCN transduced with the Kv4.1-targeted shRNA-expressing (*n* = 16), compared with the nontargeted shRNA-expressing (*n* = 14), AAV8.

To determine whether the loss of Kv4.1 also plays a role in setting the period of clock gene expression, we measured bioluminescence in *Per2^Luc^* SCN explants transduced with the Kv4.1-targeted shRNA- or the nontargeted shRNA-expressing AAV8. As illustrated in Figure 5*A*, all SCN explants examined exhibited high amplitude circadian rhythms for at least 5 d. The mean period of Per2^Luc^ expression in SCN explants expressing the Kv4.1*-*targeted shRNA (23.7 ± 0.1 h) was significantly (*p* < 0.004) shorter than in the SCN explants expressing the nontargeted shRNA (24.6 ± 0.1 h; Fig. 5*B*)^l^. The peak-to-trough amplitudes of the oscillations, however, were not significantly (*p* > 0.05) different in Kv4.1-targeted shRNA-expressing (332144 ± 140590 counts/10 min) and nontargeted shRNA-expressing (383865 ± 72192 counts/10 min) SCN explants. The acute knockdown of Kv4.1, therefore, markedly affected the circadian period, but not the amplitude, of clock gene (Per2^Luc^) expression in the SCN (see Discussion).

### Knockdown of Kv4.1 shortens the circadian period of wheel-running activity

**Figure 6.**
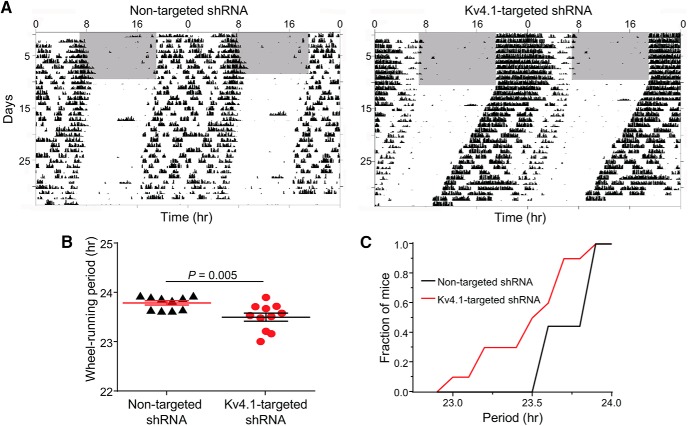
Acute knockdown of Kv4.1 shortens the circadian period of locomotor behavior. ***A***, Representative recordings of the wheel-running activity of mice that received bilateral SCN injections of either the nontargeted shRNA-expressing (left) or the Kv4.1-targeted shRNA-expressing (right) AAV8. Over 50 consecutive days, the activity of each mouse was recorded in different light-dark (indicated by the gray and white backgrounds, respectively) conditions. Each line shows wheel revolutions per minute over a 48-h period. ***B***, The mean ± SEM circadian period of locomotor activity in DD was significantly (*p* < 0.001; Student’s *t* test) shorter in mice expressing the Kv4.1-targeted shRNA (*n* = 11), compared with mice expressing the nontargeted shRNA (*n* = 10). ***C***, Cumulative distribution plots of the intrinsic periods of locomotive activity also revealed that ∼50% of mice transduced with the Kv4.1-targeted shRNA-expressing AAV8 had significantly (*p* < 0.0001, two-sample Kolmogorov-Smirnov test) shorter periods in DD than mice infected with the nontargeted shRNA-expressing AAV8.

Representative recordings of wheel-running activity in mice following injection of the Kv4.1-targeted or the nontargeted shRNA-expressing AAV8 are illustrated in Figure 6*A*. In a standard 12:12 h LD cycle, mice injected with the Kv4.1-targeted shRNA- or the nontargeted shRNA-expressing AAV8 entrained normally, showing no differences in the time of daily activity onset (Kv4.1-targeted shRNA: 18:57 ± 0.1 h and nontargeted shRNA: 18:29 ± 0.3 h). In DD, the circadian period of wheel-running was significantly (*p* < 0.005) shorter in mice injected with the Kv4.1-targeted shRNA-expressing AAV8 (23.5 ± 0.1 h) than in mice receiving the nontargeted shRNA-expressing AAV8 (23.8 ± 0.1 h; Fig. 6*B*)^m^. In addition, ∼50% of the mice expressing the Kv4.1-targeted shRNA had shorter periods than any of the mice expressing the nontargeted shRNA (Fig. 6*C*)^n^. Total activity during both the subjective day and night, however, was unaffected by the knockdown of Kv4.1 in the SCN (Kv4.1-targeted shRNA: 19800 ± 1603 counts/10 min during the active phase and 502 ± 180 counts/10 min during the inactive phase; nontargeted shRNA: 19750 ± 1653 counts/10 min during the active phase and 634 ± 200 counts/10 min during the inactive phase). Furthermore, the relative amount of time spent running-to-resting each day (α/ρ) was not significantly different in Kv4.1-targeted shRNA-expressing (2.4 ± 0.4 h) and nontargeted shRNA-expressing (2.1 ± 0.2 h) mice.

## Discussion

The results presented here provided the first direct demonstration of a functional role for Kv4.1-encoded I_A_ channels in the mammalian brain. Although *Kcnd1* (Kv4.1) was the first member of the Kv4 subfamily cloned (Pak et al., 1991), it is expressed at much lower levels than the other members of this subfamily, *Kcnd2* (Kv4.2) and *Kcnd3* (Kv4.3) in most brain regions (Serodio et al., 1996) and most of the functional studies have focused on defining the properties and physiologic roles of Kv4.2- and Kv4.3-encoded I_A_ channels (Kim et al., 2005; Chen et al., 2006; Andrasfalvy et al., 2008; Carrasquillo et al., 2012). In the striatum (Song et al., 1998) and amygdala (Dabrowska and Rainnie, 2010) and in dorsal root ganglion neurons (Phuket and Covarrubias, 2009), where *Kcnd1* expression is high, roles for Kv4.1-encoded I_A_ channels have been inferred, although the molecular and/or pharmacological approaches used targeted all members of the Kv4-subfamily and were not selective for Kv4.1. In the experiments here, we used a Kv4.1-selective shRNA to show directly that Kv4.1 contributes to the generation of I_A_ in adult mouse SCN neurons and, in addition, that Kv4.1-encoded I_A_ channels function in the regulation of the active and passive membrane properties of SCN neurons as well as daily rhythms in repetitive firing, clock gene expression and locomotor behavior.

### Kv4.1 regulates circadian periodicity

Acute *in vivo* knockdown of Kv4.1 expression in adult mouse SCN neurons significantly shortened the circadian period of electrical activity, clock gene expression, and locomotor behavior. These observations demonstrate that changes in membrane excitability feedback to the molecular clock, resulting in alterations in circadian rhythms and behavioral outputs. This conclusion is consistent with several previous studies using pharmacological, molecular genetic, and/or optogenetic approaches to manipulate the excitability of SCN neurons *in vivo* (Lundkvist et al., 2005; Granados-Fuentes et al., 2012; Granados-Fuentes et al., 2015; Jones et al., 2015). Indeed, it has been shown, using targeted deletion strategies that Kv1.4- and Kv4.2-, but not Kv4.3-, encoded I_A_ channels also regulate SCN excitability and circadian rhythms in behavior and clock gene expression (Granados-Fuentes et al., 2012; Granados-Fuentes et al., 2015). Daily changes in the excitability of SCN neurons, mediated at least in part by I_A_ channels, therefore, lie both on the input to, and output from, the molecular clock.

### Kv4.1 regulates intrinsic firing properties in SCN neurons

Daily rhythms in the intrinsic membrane properties and the spontaneous firing rates of mammalian SCN neurons have been studied extensively (Green and Gillette, 1982; de Jeu et al., 1998; Meredith et al., 2006; Colwell, 2011). Although there are some differences in absolute values reported, on average, daytime repetitive firing rates are ∼5 Hz, whereas, at night, repetitive firing rates are ∼1 Hz and a number of K_v_ (Kudo et al., 2011) and voltage-gated Na^+^ (Jackson et al., 2004; Paul et al., 2016), as well as Ca^2+^-dependent K^+^ (Meredith et al., 2006), channels have been shown to contribute to the regulation of repetitive firing rates in SCN neurons. In addition, however, the R_in_ of SCN neurons are higher during the day than at night, findings interpreted as suggesting that the observed day-night differences in repetitive firing rates are mediated, at least in part, by diurnal changes in subthreshold K^+^ conductance(s) (Kuhlman and McMahon, 2004; Flourakis et al., 2015). Unlike many other K_v_ currents, A-type channels, in addition to regulating repetitive firing rates, function in the subthreshold range of membrane potentials in some neuronal cell-types (Kim et al., 2005; Carrasquillo et al., 2012). As illustrated in Figure 2*G*, a clear I_A_ window current is observed over the voltage range (−35 to −55 mV) of V_r_ typically observed of SCN neurons (Table 1), indicating that some I_A_ channels are open at these potentials. The increases in R_in_ and repetitive firing rates observed here in the targeted shRNA expressing cells reveals a role for Kv4.1, either alone or with other Kv4 subfamily members, in the generation of the critical I_A_ channels.

Expression of the Kv4.1-targeted shRNA significantly decreased ISIs and increased repetitive firing rates in SCN neurons during the day and at night, suggesting that Kv4.1-encoded I_A_ channels function by acting as a “brake” to control repetitive firing rates at all times throughout the day. In recordings from dispersed SCN neurons, we also observed a significant increase in repetitive firing rates during the peak (subjective day) and the trough (subjective night) of electrical activity over multiple days.

The results presented here also revealed that the acute knockdown of Kv4.1 had differential effects on the daytime and nighttime R_in_ and repetitive firing rates in SCN neurons. During the day, the mean repetitive firing rate of SCN neurons expressing the Kv4.1-targeted shRNA was approximately twice the rate measured in control SCN neurons expressing the nontargeted shRNA. At night, however, the mean repetitive firing rate of Kv4.1-targeted shRNA-expressing SCN neurons was three-fold higher than in control, nontargeted shRNA-expressing cells. The effects of Kv4.1 knockdown on the R_in_ of SCN neurons and on the amplitudes of I_A_ were also greater at night than during the day. These observations suggest that the expression of Kv4.1 may be under circadian control. Data deposited in the CircaDB circadian expression profiles database (http://circadb.hogeneschlab.org), however, indicate that *Kcnd1* transcript expression does not vary with time of day. It has also been reported that the expression of the Kv4.1 protein is similar at night and during the day (Itri et al., 2010). Unfortunately, owing to the lack of availability of a Kv4.1-targeted deletion (Kv4.1^−/−^) mouse line, the antibody used to probe Kv4.1 protein expression in this study has not been validated to date. Additional studies focused on examining Kv4.1 protein expression will need to be done when a Kv4.1^−/−^ mouse line and validated anti-Kv4.1 antibodies become availiable. It is certainly also possible that day-night differences in the functional expression of Kv4.1-encoded I_A_ could be produced by rhythmic changes in posttranslational modifications of the Kv4.1 α-subunit or of accessory subunits, as recently suggested for circadian regulation of the persistent Na^+^ current in SCN neurons (Paul et al., 2016). The availability of validated anti-Kv4.1 antibodies will also make it possible to test these hypotheses directly.

## Synthesis

Reviewing Editor: Rae Silver, Columbia University Decisions are customarily a result of the Reviewing Editor and the peer reviewers coming together and discussing their recommendations until a consensus is reached. When revisions are invited, a fact-based synthesis statement explaining their decision and outlining what is needed to prepare a revision will be listed below.

Summary: Suprachiasmatic nucleus neurons fire action potentials in a circadian pattern with faster firing frequencies observed during the day then during the night. The biophysical mechanisms underlying this firing pattern are not known, but are believed to be due to circadian regulation of ion channel activity. The current manuscript examines the role of the Kv4.1 channel subunit in regulating the activity of action potential firing in SCN neurons. The methods and experimental design are very clearly described. The experimental strategy examines the effect of Kv4.1 knockdown on the circadian system at multiple levels.

Major Comments: In its current state the manuscript does not provide any insights into why/how the Kv4.1 channel subunit is unique in regulating the activity of suprachiasmatic nucleus neurons. A number of potassium channels when knocked out produce alterations in SCN neuronal activity and circadian rhythms. The Discussion section of the results is not in-depth and does not provide any insights into the possible mechanisms underlying the changes produced by the Kv4.1 knockdown.

There is no data presented indicating the magnitude of the reduction in the Kv4.1 in the organotypic cultures or in vivo. The Western blots in Figure 1 are from the cell lines used to characterize the shRNA. The authors are assuming that the same level of subunit reduction will occur in the brain tissue as in the cell lin¬¬¬e. The data would be significantly stronger with the addition of this data.

Specific comments

1. The membrane input resistance is significantly changed by Kv4.1 knockdown. Since the Kv4.1 current is voltage sensitive it is not clear how knocking down Kv4.1 would significantly change the input resistance- a point that should be considered in the Discussion.

2. The Kv4.1 knockdown produced only a 35-50% reduction in the current amplitude. Were some of the effects potentially mediated by changes in the activation or inactivation kinetics of the remaining IA currents?

3. In Figure 2 it would be useful to show expanded examples of action potentials to show the effects of Kv4.1 knockdown. The IA current plays an important role in action potential repolarization. Is the rate of repolarization altered in the Kv4.1 knockdown mice?

4. The use of a Student's t-test to test for a difference of means is not appropriate. The data presented in Fig. 3D shows a lack of balance and skewness that goes in opposite directions. The authors note this in the legend for 3C. In addition, the two data sets may have different variances and they have significantly different numbers of neurons (35 vs 63). A more appropriate test might be a logistic regression analysis.

5. Figure 4 has a lot of information but is cluttered and somewhat redundant. Since the calibration bars are all the same only one bar seems necessary. Similarly since the current steps are all the same only one example is needed. The lowest and highest voltage values could be added to the steps for clarity.

6. A style comment. The data shown in Figure 4 should be shown after Figure 1. The importance of knocking down Kv4.1 will depend on the magnitude of the reduction of the IA current. These data should be presented earlier in the Results.

7. Regarding the statement on page 13: “approximately 90% of the mice expressing the Kv4.1-targeted shRNA had shorter periods than any of the mice expressing the non-targeted shRNA.” Is this correct? Examination of Figure 6C indicates that 50% of the experimental Kv4.1-targeted shRNA mice have shorter periods than ANY on the control non-targeted shRNA ones.

8. In the discussion (page 15) it is stated that “the increase in daytime and nighttime repetitive firing rates in SCN cells expressing the Kv4.1-targeted shRNA ”are correlated“ with shortening of interspike intervals.” Is this actually a correlation? The increase in firing rate is achieved by decreasing the interspike interval.

9. The Discussion section does not go into any depth on the significance of the data presented. In particular there is no discussion of how the removal of this subunit would produce the action potential frequency alterations observed. An effort should be made to explain how KV4 in general and KV4.1 in particular could regulate SCN firing rate.
